# Correction to: IL‑15 superagonist N‑803 improves IFNγ production and killing of leukemia and ovarian cancer cells by CD34+ progenitor‑derived NK cells

**DOI:** 10.1007/s00262-021-03049-5

**Published:** 2021-09-15

**Authors:** J. M. R. Van der Meer, R. J. A. Maas, K. Guldevall, K. Klarenaar, P. K. J. D. De Jonge, J. S. Hoogstad-van Evert, A. B. van der Waart, J. Cany, J. T. Safrit, J. H. Lee, E. Wagena, P. Friedl, B. Önfelt, L. F. Massuger, N. P. M. Schaap, J. H. Jansen, W. Hobo, H. Dolstra

**Affiliations:** 1 Department of Laboratory Medicine, Laboratory of Hematology, Radboud University Medical Center, Radboud Institute for Molecular Life Sciences, Geert Grooteplein Zuid 8, P.O. Box 9101, 6500 HB Nijmegen, The Netherlands; 2 Department of Applied Physics, Science for Life Laboratory, KTH - Royal Institute of Technology, Stockholm, Sweden; 3 NantKwest, Culver City, CA USA; 4 ImmunityBio, Culver City, CA USA; 5 Department of Cell Biology, Radboud Institute for Molecular Life Sciences, Nijmegen, The Netherlands; 6 David H. Koch Center for Applied Genitourinary Cancers, The University of Texas MD Anderson Cancer Center, Houston, TX USA; 7 Cancer Genomics Center, Utrecht, The Netherlands; 8 Department of Obstetrics and Gynecology, Radboud University Medical Center, Nijmegen, the Netherlands; 9 Department of Hematology, Radboud University Medical Center, Nijmegen, The Netherlands

## Correction to: Cancer Immunology, Immunotherapy (2021) 70:1305–1321 10.1007/s00262-020-02749-8

Unfortunately, the given name and family name of the sixth author was incorrectly tagged in the xml data, therefore it is abbreviated wrongly as ‘‘Evert, JSH’’ in Pubmed. The correct given name is J.S. and family name is Hoogstad-van Evert.

